# Tropospheric Warming Over The Past Two Decades

**DOI:** 10.1038/s41598-017-02520-7

**Published:** 2017-05-24

**Authors:** Benjamin D. Santer, Susan Solomon, Frank J. Wentz, Qiang Fu, Stephen Po-Chedley, Carl Mears, Jeffrey F. Painter, Céline Bonfils

**Affiliations:** 1 Program for Climate Model Diagnosis and Intercomparison (PCMDI), Lawrence Livermore National Laboratory, Livermore, CA 94550 USA; 2 Massachusetts Institute of Technology, Earth, Atmospheric, and Planetary Sciences, Cambridge, MA 02139 USA; 3 Remote Sensing Systems, Santa Rosa, CA 95401 USA; 4 Dept. of Atmospheric Sciences, University of Washington, Seattle, WA 98195 USA

## Abstract

Satellite temperature measurements do not support the recent claim of a “leveling off of warming” over the past two decades. Tropospheric warming trends over recent 20-year periods are always significantly larger (at the 10% level or better) than model estimates of 20-year trends arising from natural internal variability. Over the full 38-year period of the satellite record, the separation between observed warming and internal variability estimates is even clearer. In two out of three recent satellite datasets, the tropospheric warming from 1979 to 2016 is unprecedented relative to internally generated temperature trends on the 38-year timescale.

## Introduction

After a recent Senate confirmation hearing, Scott Pruitt – the new Administrator of the U.S. Environmental Protection Agency – received a written question regarding observed warming estimates. In response, Mr. Pruitt claimed that “over the past two decades satellite data indicates there has been a leveling off of warming”^1^. We test this claim here. In the following, we assume the satellite data referred to by Mr. Pruitt are measurements of the temperature of the lowest layer of the atmosphere (the troposphere). These measurements were the focus of recent Congressional testimony^2^. We update and extend the analysis in ref. 3 using satellite temperature data spanning the period from January 1979 to December 2016.

Since late 1978, satellite microwave sounders have monitored the microwave emissions of oxygen molecules. Emissions are proportional to the temperature of different atmospheric layers, and require adjustments for known problems associated with satellite orbital drift and instrument calibration^4–6^. Satellite estimates of global changes in the temperature of the mid- to upper troposphere (TMT) are currently available from Remote Sensing Systems (RSS), the Center for Satellite Applications and Research (STAR), and the University of Alabama at Huntsville (UAH). All three groups provide older and more recent TMT datasets^4, 5, 7^. The newer, more reliable datasets are the primary focus here.

**Figure 1:**
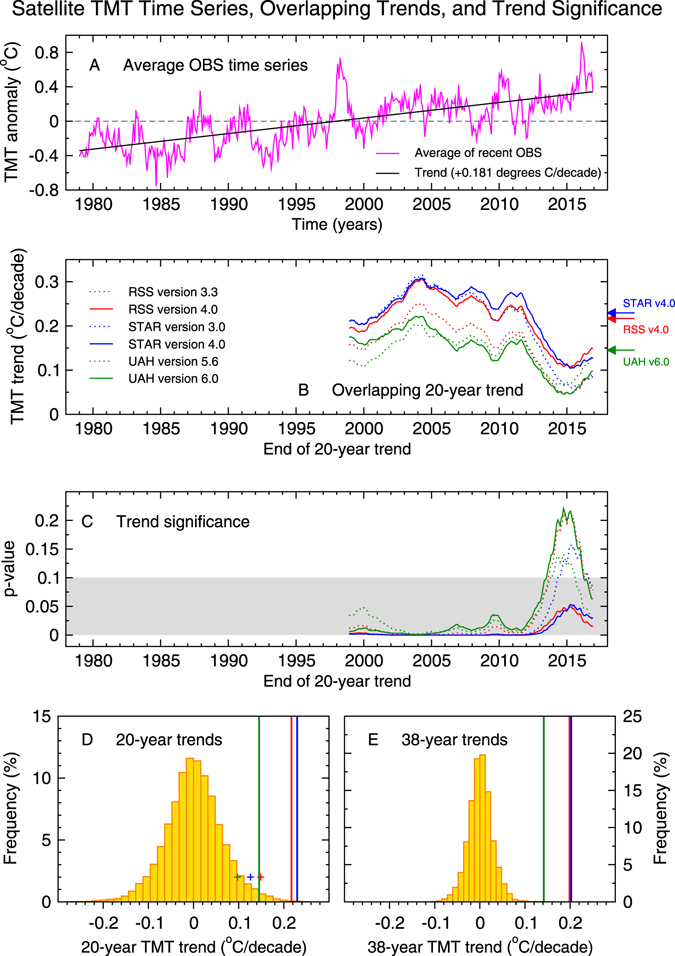
Evaluation of claimed “leveling off” of warming in satellite temperature data. Results are for monthly-mean anomalies in the temperature of the mid- to upper troposphere (TMT), corrected for stratospheric cooling^8^ and spatially averaged over 82.5°N–82.5°S. The average of the latest satellite dataset versions (RSS v4.0, STAR v4.0, and UAH v6.0) has a warming trend of 0.181 °C/decade over the 456-month period from January 1979 to December 2016 (panel A). Maximally overlapping 20-year (240-month) trends in the six individual satellite TMT time series are plotted on the end month of the trend-fitting period (panel B). The *p*-values for these trends (panel C) are for tests of the null hypothesis that observed tropospheric warming could be due to natural internal variability alone^3^. The grey shaded box is the rejection region (at a stipulated 10% significance level) for the null hypothesis. The *p*-value calculations rely on estimates of the multi-decadal internal variability of the climate system from model pre-industrial control runs. These simulations have no year-to-year changes in natural or human external forcings. The sampling distributions of control run TMT trends on 20-year and 38-year timescales (the orange histograms in panels D and E, respectively) are based on results from 36 different models. The symbols (plus signs) in panel D are the final 20-year trends from panel B. Bold vertical lines in panel D are the averages of the overlapping 20-year trends in panel B (see arrows to the right of panel B). Vertical lines in panel E are observed trends over the full 456-month satellite record. Results in D and E are for the latest satellite dataset versions only. Full analysis details are in ref. 3 and the Methods section.

Satellite TMT measurements include a contribution from the cooling stratosphere. To study warming of the troposphere, we used a standard regression method^8–11^ to remove the stratospheric cooling contribution to TMT (see Methods). The corrected TMT data show pronounced tropospheric warming (Fig. 1A). In the most recent versions of the RSS, STAR, and UAH datasets, the TMT trend over the full 38-year period of the satellite record is 0.199 °C, 0.202 °C, and 0.142 °C per decade (respectively).

To evaluate the claim that satellite data show “leveling off” of warming over the past two decades, we examine all possible 20-year periods of satellite TMT records. We calculate linear temperature trends for each 20-year period, and then determine whether the observed TMT trends are significantly larger than the 20-year trends arising from natural processes internal to the climate system^3^. Estimates of natural internal variability are based on results from 36 different climate models (see Methods and Supplementary Information).

## Results

In each of the six satellite datasets, all 20-year TMT trends are positive, irrespective of the trend start date (Fig. 1B). The specific period of “the past two decades” yields 20-year TMT trends that have not “leveled off”. As expected, there are multi-decadal changes in trend size^12, 13^. Recent 20-year trends are smaller than most of the earlier 20-year trend values. This is due to the combined effects of multiple factors: the anomalous warmth at the beginning of the last 20 years (arising from a large El Niño event in 1997/98), the shift from a warm phase to a cold phase of the Interdecadal Pacific Oscillation in the late 1990s^14–17^, changes in other modes of internal variability^18–20^, a succession of moderate volcanic eruptions in the early 21st century^21–23^, a long and low minimum in solar output during the last solar cycle^24^, and an increase in anthropogenic sulphate pollution^25, 26^.

Figure 1C provides information on whether observed TMT trends show unusually large warming relative to the estimated warming trends caused by natural internal climate variability. Two features are noteworthy. First, we find that significant 20-year tropospheric warming trends are a commonplace occurrence during the satellite era. Second, despite their smaller size, warming trends over the last 20 years (January 1997 to December 2016) are significantly larger, at the 10% level or better, than estimates of 20-year trends arising from natural internal variability (Fig. 1C,D). This holds for all six satellite datasets. In the latest versions of the RSS, STAR, and UAH TMT data, the probability that internal variability could produce warming exceeding that observed over the last 20 years is only 1.6%, 3.1%, and 6.3% (respectively). These probabilities decrease markedly if the averages of all individual 20-year trends are considered (see vertical lines in Fig. 1D).

The unusual size of observed tropospheric warming becomes even clearer for the full 38-year period of TMT measurements. Over 1979 to 2016, global warming of the troposphere far exceeds current estimates of natural internal climate variability (Fig. 1E). TMT trends in the latest versions of the RSS, STAR, and UAH datasets are (respectively) 7.50, 7.64, and 5.35 standard deviations removed from the mean of the distribution of unforced 38-year TMT trends. The probabilities associated with these numbers are miniscule. In fact, the tropospheric warming trends in versions 4.0 of the RSS and STAR data are unprecedented – they are not exceeded by any of the 212,808 unforced TMT trends in the distribution shown in Fig. 1E. In version 6.0 of the UAH data, only 16 of the 212,808 unforced trends are larger than the observed TMT trend. To plausibly explain the observed tropospheric warming by natural internal variability would require that the model results in Fig. 1E underestimate real-world internal variability by a factor of 2.5 or more. There is no evidence of a systematic model error of this size^12, 27, 28^ (see Methods).

## Summary

Satellite temperature measurements do not support the claim of a “leveling off of warming” over the past two decades^1^. They are also inconsistent with a similar claim^2^ (see Supplementary Figure S1). Trend assessments over short, 1–2 decade-long periods of time are often sensitive to small changes in the trend start date^3^. More reliable estimates of underlying temperature changes are obtained by averaging over all possible short-term trends or by considering longer analysis periods.

When examined over the full period of record, long-term tropospheric warming far exceeds current estimates of natural internal climate variability (Fig. 1E). Our results support and strengthen previous findings of a large human-caused contribution to warming^29–32^. Studies involving patterns of tropospheric temperature change (rather than the global averages considered here) yield even stronger evidence of a human fingerprint in the thermal structure of the atmosphere^27, 33–35^. The recent focus on satellite temperature data in political discourse^1, 2^ provides an opportunity to highlight this fingerprint evidence, and underscores the importance of continued satellite-based monitoring of Earth’s climate.

## Methods

### Satellite atmospheric temperature data

We used satellite estimates of atmospheric temperature produced by RSS^4^, STAR^5^, and UAH^7^. All three groups provide satellite measurements of the temperatures of the mid- to upper troposphere (TMT) and the lower stratosphere (TLS). Our focus here is on assessing the significance of observed trends in TMT. TLS is required for correcting TMT for the influence it receives from stratospheric cooling.

Each group provides the most recent version and the previous version of their datasets. The versions available are: 3.3 and 4.0 (RSS), 3.0 and 4.0 (STAR), and 5.6 and 6.0 (UAH). Satellite datasets are in the form of monthly means on 2.5° × 2.5° latitude/longitude grids. At the time this analysis was performed, temperature data were available for the 456-month period from January 1979 to December 2016.

There are differences in the spatial coverage of the satellite temperature data produced by the three groups. While UAH TLS and TMT datasets have global coverage, areas poleward of 87.5° (82.5°) are excluded from STAR (RSS). To avoid any impact of spatial coverage differences on trend comparisons, we calculated all near-global averages of actual and synthetic satellite temperatures over the area of common coverage in the RSS, UAH, and STAR datasets (82.5°N to 82.5°S).

### Method used for correcting TMT data

Trends in TMT estimated from microwave sounders receive a substantial contribution from the cooling of the lower stratosphere^8–11^. In ref. 8, a regression-based approach was developed for removing the bulk of this stratospheric cooling component of TMT. This method has been validated with both observed and model atmospheric temperature data^9, 36, 37^. Correction was performed at each observational and model grid-point. Corrected grid-point data were then spatially averaged over 82.5°N–82.5°S. Further details of the correction method are provided in the Supplementary Information.

### Details of model output

We used model output from phase 5 of the Coupled Model Intercomparison Project^38^ (CMIP5). The simulations analyzed here were contributed by 18 different research groups (see Supplementary Table S1). Our focus was on pre-industrial control runs with no changes in external influences on climate, which provide estimates of the natural internal variability of the climate system (see Supplementary Table S2).

To compare satellite-derived atmospheric temperature trends with model estimates of trends arising from natural internal variability, we calculate synthetic TMT and TLS from CMIP5 control runs. This calculation relies on a local weighting function method developed at RSS. At each model grid-point, simulated temperature profiles were convolved with local weighting functions. Local weights depend on the grid-point surface pressure, the surface type (land or ocean), and the selected layer-average temperature (TMT or TLS).

### Statistical analysis

We use model estimates of natural internal variability to evaluate the statistical significance of trends in the observed temperature time series *T*_*o*_(*k*, *t*), where *k* and *t* are (respectively) indices over the number of satellite TMT datasets and the time in months. Internal variability estimates are obtained from CMIP5 control runs. Rather than focusing on one specific period, we analyze maximally overlapping 20-year trends in *T*_*o*_(*k*, *t*). “Maximally overlapping” indicates that an 20-year sliding window is being used for trend calculations. This window advances in increments of one month until the end of the current window reaches the final month of the satellite or control run TMT time series.

Anomalies in the satellite observations are defined relative to climatological monthly means calculated over the 38-year period from January 1979 to December 2016. Control run anomalies are with respect to climatological monthly means over the full length of each model’s control integration.

We assess trend significance using weighted *p*-values, which account for inter-model differences in control run length^3^.

The weighted *p*-value, $\overline{{p}_{c}}(i,k)^{\prime}$, is defined as:

1 $$\overline{{p}_{c}}(i,k)^{\prime} =\sum _{j=1}^{{N}_{model}}{p}_{c}(i,j,k)/{N}_{model}$$

$$i=\mathrm{1,}\ldots ,{N}_{o};j=\mathrm{1,}\ldots ,{N}_{model};k=\mathrm{1,}\ldots ,{N}_{sat}$$

where the index *i* is over *N*_*o*_, the number of maximally overlapping 20-year trends in *T*_*o*_(*k*, *t*), and the index *j* spans *N*_*model*_, the number of model control runs (which is 36 here). The sample size *N*_*sat*_ is the total number of satellite datasets. Here, *N*_*sat*_ = 6, and *N*_*o*_ = 217 for 20-year trends.

The individual *p*_*c*_(*i*, *j*, *k*) values for each model pre-industrial control run are calculated as follows:

2 $${p}_{c}(i,j,k)={K}_{c}(i,j,k)\,/{N}_{c}(j)$$

$$i=\mathrm{1,}\ldots ,{N}_{o};j=\mathrm{1,}\ldots ,{N}_{model};k=\mathrm{1,}\ldots ,{N}_{sat}$$

where the summation variable *K*_*c*_(*i*, *j*, *k*) is the number of 20-year trends in each model control run that are larger than *b*_*o*_(*i*, *k*), the current 20-year trend in *T*_*o*_(*k*, *t*). The sample size *N*_c_(*j*) is the number of maximally overlapping 20-year trends in the *j* ^*th*^ control run. Further information on the statistical notation and analysis is given in the Supplementary Information.

### Sensitivity of results to model variability errors

The credibility of our trend significance results rests on the assumption that model control runs yield reliable estimates of internal variability on the timescales considered here (20 years in Fig. 1C and D, 38 years in Fig. 1E, and 18 years in Supplementary Fig. 1C and D). On these multi-decadal timescales, it is not feasible to use the single realization of the observed 38-year satellite TMT record to evaluate how reliably models capture “observed” internal variability. The primary difficulty is that observed temperature records are simultaneously influenced by both internal variability (operating on a wide range of different space and timescales) and multiple external forcings. Unambiguous partitioning of observational temperature records into internally generated and externally forced components is an aspirational goal, but not attainable in practice. All model-versus-observed internal variability comparisons are affected by the uncertainties involved in isolating multi-decadal internal variability from observational climate records^27^.

Other approaches must therefore be employed to enhance confidence in the reliability of model variability on 18- to 38-year timescales, such as variability comparisons involving longer SST and land + ocean surface temperature records^12, 28^. The latter work shows no evidence that models systematically underestimate observed variability on multi-decadal timescales – see, *e.g*., Fig. 4 in ref. 28. The same applies to model-versus-data variability comparisons on shorter timescales of roughly 10 years^27^.

## Electronic supplementary material

Supplementary Information
