# Supplementary material for: Tropospheric Warming Over The Past Two Decades
Source: Sci Rep. 2017 May 24;7:2336. doi: 10.1038/s41598-017-02520-7 (PMC5443760; doi:10.1038/s41598-017-02520-7)
Supplement: Supplementary file 1 — Supplementary Information [file 41598_2017_2520_MOESM1_ESM.pdf]

# Supplementary Information

# Tropospheric Warming Over The Past Two Decades

Benjamin D. Santer[1,*], Susan Solomon[2], Frank J. Wentz[3], Qiang Fu[4], Stephen Po-Chedley[1], Carl Mears[3], Jeffrey F. Painter[1], & Céline Bonfils[1]

[1]Program for Climate Model Diagnosis and Intercomparison (PCMDI), Lawrence Livermore National Laboratory, Livermore, CA 94550, USA.

[2]Massachusetts Institute of Technology, Earth, Atmospheric, and Planetary Sciences, Cambridge, MA 02139, USA.

[3]Remote Sensing Systems, Santa Rosa, CA 95401, USA.

[4]Dept. of Atmospheric Sciences, University of Washington, Seattle, WA 98195, USA.

Corresponding author's email: santer1@llnl.gov

# Satellite temperature data: Additional information

Since late 1978, microwave sounders on NOAA polar-orbiting satellites have measured the microwave emissions of oxygen molecules. Because oxygen molecules are present at all altitudes, the microwave radiance that reaches the satellite is an integral of emissions from thick layers of the atmosphere*. The observed microwave radiance, or "brightness temperature", is related to the average temperature of a broad layer of the atmosphere by a weighting function, which describes the relative contribution of each level of the atmosphere to the total radiance. The weighting function is calculated using an atmospheric radiative transfer model. The function depends both on the microwave frequency band that is observed and the angle of observation relative to Earth's surface, allowing the sounder to measure different layers in the atmosphere via the use of different frequency bands and/or different viewing angles[1,2,3].

We used satellite estimates of atmospheric temperature change produced by three different research groups:

1. Remote Sensing Systems in Santa Rosa, California (RSS)[1,4].
2. The Center for Satellite Applications and Research, NOAA/National Environmental Satellite, Data, and Information Service, College Park, Maryland (STAR)[2,5,6].

*Satellite estimates of the temperature of tropospheric layers also receive a small contribution from the temperature at Earth's surface.

3. The University of Alabama at Huntsville (UAH)[7].

All three groups provide satellite estimates of the temperature of the mid- to upper troposphere (TMT).[†] Trends in TMT are the focus of the current study. RSS, UAH, and STAR also produce satellite measurements of the temperature of the lower stratosphere (TLS). TLS is required for correcting TMT for the influence it receives from stratospheric cooling. The approximate altitude ranges and pressure level boundaries for TMT and TLS are given in Table 2 of ref. 8.

The construction of a long-term data record from satellite data requires that measurements from over a dozen satellites are intercalibrated and merged. The three groups use different methods to account for: 1) the effects of inter-satellite differences in the MSU calibration; 2) calibration drift as the physical temperature of the satellite varies; 3) changes in the design and measurement frequencies of the MSU instrument itself; and 4) the effects of drifts in the time of day that the measurements are made.

## Correcting TMT for stratospheric cooling

Trends in TMT estimated from microwave sounders receive a substantial contribution from the cooling of the lower stratosphere[9,10,11,12]. In ref. 9, a regression-based ap-

[†]The University of Washington (UW) also produces a TMT dataset, but this is available for the tropics only[3]. Since the interest here is in global-scale changes in TMT, we did not analyze UW TMT data for the present study.

proach was developed for removing the bulk of this stratospheric cooling component of TMT. In the Supplementary Information, we refer to this "corrected" version‡ of TMT as $\text{TMT}_{cr}$. The main text discusses corrected TMT only, and does not use the subscript *cr* to identify corrected TMT.

The correction method appiled in ref. 9 has been validated with both observed and model atmospheric temperature data[10,13,14]. Correction was performed locally, at each observational and model grid-point. Corrected grid-point data were then spatially averaged over 82.5°N-82.5°S.

For calculating tropical averages of $\text{TMT}_{cr}$, ref. 11 used:

$$\text{TMT}_{cr} = a_{24}\text{TMT} + (1 - a_{24})\text{TLS} \quad (1)$$

where $a_{24} = 1.1$. Subsequent analyses of tropical data in ref. 12 obtained very similar estimates§ of $a_{24}$. For the near-global domain considered here, lower stratospheric cooling makes a larger contribution to TMT trends¶, so $a_{24}$ is larger[9,12]. In refs. 9 and 12, $a_{24} \approx 1.15$ was applied directly to near-global averages of TMT and TLS. Since

‡In other publications[3,11], $\text{TMT}_{cr}$ is designated as TTT (the temperature of the tropical troposphere) or as $\text{T}_{24}$ (since it is generated using brightness temperatures estimated with the emissions measurements obtained from channels 2 and 4 of microwave sounders).

§See Table 1 in 12.

¶This is due to two effects: the tropopause is lower at mid- to high latitudes than in the tropics, and stratospheric cooling over the satellite era is larger at high latitudes than in the tropics[15].

we are performing corrections on local (grid-point) data, we used $a_{24} = 1.1$ between 30°N and 30°S, and $a_{24} = 1.2$ poleward of 30°. This is approximately equivalent to use of the $a_{24} = 1.15$ for globally-averaged data.

Finally, we note that model and observational temperature data were processed in exactly the same way – *i.e.*, model-versus-observed differences in corrected TMT trends in Figs. 1D and E of the main text (and in Supplementary Fig. S1D) are not attributable to differences in the applied regression coefficients.

In calculating corrected TMT from UAH TLS and TMT data, we did not 'mix' different versions of the UAH datasets: *i.e.*, version 5.6 of UAH $\text{TMT}_{cr}$ was computed with version 5.6 of UAH TLS and TMT data, and version 6.0 of UAH $\text{TMT}_{cr}$ was computed with version 6.0 of UAH TLS and TMT data. The same holds for the STAR corrected TMT data: version 3.0 (4.0) of STAR $\text{TMT}_{cr}$ was calculated with version 3.0 (4.0) of STAR TLS and TMT data.

For RSS, version 3.3 of $\text{TMT}_{cr}$ was calculated with version 3.3 of RSS TLS and TMT data. Version 4.0 of RSS $\text{TMT}_{cr}$ relied on version 4.0 of RSS TMT and version 3.3 of RSS TLS (since version 4.0 of RSS TLS is not yet available). The residual errors that were corrected in the transition from version 3.3 to version 4.0 of the RSS TMT data are unlikely to have pronounced impact on TLS, so the inconsistency in the TMT and TLS versions used to generate version 4.0 of the RSS $\text{TMT}_{cr}$ data is not important[1].

## Model output: Additional information

We used model output from phase 5 of the Coupled Model Intercomparison Project (CMIP5)[16]. A full list of modeling groups participating in CMIP5 is given at http://cmip-pcmdi.llnl.gov/cmip5/docs/CMIP5_modeling_groups.pdf. The simulations analyzed here were contributed by 18 different research groups (see Supplementary Table S1). Our focus was on pre-industrial control runs with no changes in external influences on climate, which provide estimates of the natural internal variability of the climate system (see Supplementary Table S2).

### Calculation of synthetic satellite temperatures

In many previous comparisons of modeled and observed atmospheric temperature trends, a global-mean weighting function was convolved with the atmospheric temperature profiles at each model grid-point. There is a different global-mean weighting function for each atmospheric layer of interest. Here, we use a local weighting function method developed at RSS. At each model grid-point, simulated temperature profiles were convolved with local weighting functions. Local weights depend on the grid-point surface pressure, the surface type (land or ocean), and the selected layer-average temperature (TLS or TMT). This method provides more accurate estimates of synthetic satellite temperatures, particularly over high elevation regions[15].

### Treatment of GISS-E2-H and GISS-E2-R models

In the `GISS-E2-H` and `GISS-E2-R` models, the same atmospheric GCM is coupled to different ocean models. In turn, each of these two coupled models provides control run simulation output for model versions with different treatment of aerosol and ozone[17,18]. For `GISS-E2-H`, synthetic MSU temperatures were available from three separate control runs (`p1`, `p2`, and `p3`). For `GISS-E2-R`, synthetic MSU temperatures were available from only two control runs (`p1` and `p2`; see Supplementary Table S2).

In calculating the "weighted" $p$-values shown in Fig. 1C and Supplementary Figure S1C, it was necessary to decide whether atmospheric temperatures from these individual model versions should be treated as different realizations of internal variability performed with a similar physical model, or as results from different models of the climate system. Since there are important differences between these model versions, we decided to treat the five different model versions (three for `GISS-E2-H` and two for `GISS-E2-R`) as five separate models.

## Statistical analysis

### Terminology

#### Abbreviations

| | |
|---|---|
| CTL | CMIP5 control run with no year-to-year changes in external forcings |

**Subscripts**

| | |
|---|---|
| $o$ | Satellite <u>o</u>bservations |
| $c$ | Output from model <u>c</u>ontrol runs |

**Indices**

| | |
|---|---|
| $i$ | Index over number of maximally overlapping trends in satellite data or model control run |
| $j$ | Index over number of model control runs |
| $k$ | Index over number of observed satellite datasets |
| $t$ | Index over time (number of months) |

**Time series**

| | |
|---|---|
| $T_o(k,t)$ | Temperature time series for $k^{th}$ observational dataset |
| $T_c(j,t)$ | Temperature time series for $j^{th}$ model control run |

**Sample sizes**

| | |
|---|---|
| $L$ | Length of trend-fitting period (years) |

| | |
|---|---|
| $N_o$ | No. of overlapping trends in observed TMT time series |
| $N_c(j)$ | No. of overlapping trends in $j^{th}$ control run |
| $N_{sat}$ | No. of satellite datasets |
| $N_{model}$ | No. of model control runs (36) |
| $N_t$ | Length of observed or control run time series (months) |

**Linear trends**

| | |
|---|---|
| $b_o(i,k)$ | Least-squares linear trend for $i^{th}$ overlapping $L$-year segment of observed TMT time series and $k^{th}$ observational dataset |
| $b_c(i,j)$ | Least-squares linear trend for $i^{th}$ overlapping $L$-year segment of control run temperature time series and $j^{th}$ control run |

**Summation variables**

| | |
|---|---|
| $K_c(i,k)$ | The number of $L$-year trends in the control run MMSD $> b_o(i,k)$, the trend in the $i^{th}$ $L$-year segment of the observed TMT time series for the $k^{th}$ observational dataset |
| $K_c(i,j,k)$ | The number of $L$-year trends in the $j^{th}$ model control run $> b_o(i,k)$ |

**Significance of observed warming trends**

$p_c(i,j,k)$ — Unweighted $p$-value for comparison of $b_o(i,k)$ and sampling distribution of maximally overlapping temperature trends from $j^{th}$ model control run

$\overline{p_c}(i,k)'$ — Weighted $p$-value, model average of $p_c(i,j,k)$

## Additional information on statistical significance issues

Our use of maximally overlapping trends has the advantage of reducing the impact of seasonal and interannual noise on underlying tropospheric temperature trends, both in $T_o(k,t)$ and in the control runs. It has the disadvantage of decreasing the statistical independence of trend samples. While non-independence of samples is an important issue in formal statistical significance testing, it is not a serious concern here. This is because $\overline{p_c}(i,k)'$ is not used as a basis for formal statistical tests. Instead, it simply provides information on whether trends in $T_o(k,t)$ are unusually large relative to model estimates of unforced trends. Furthermore, we process $T_o(k,t)$ and control run output in identical ways, with the same overlap between successive 20-year trends.

The key point is that whether we employ overlapping or non-overlapping control run trends has minimal impact on estimates of $\overline{p_c}(i,k)'$. This suggests that the sample sizes of non-overlapping trends in the CMIP5 control runs are adequate for obtaining reliable estimates of $p$-values.

# References

1. Mears, C. & Wentz, F. J. Sensitivity of satellite-derived tropospheric temperature trends to the diurnal cycle adjustment. *J. Clim.* **29**, 3629–3646 (2016).

2. Zou, C.-Z. *et al.* Recalibration of microwave sounding unit for climate studies using simultaneous nadir overpasses. *J. Geophys. Res.* **111**, DOI:10.1029/2005JD006798 (2006).

3. Po-Chedley, S., Thorsen, T. J. & Fu, Q. Removing diurnal cycle contamination in satellite-derived tropospheric temperatures: Understanding tropical tropospheric trend discrepancies. *J. Clim.* **28**, 2274–2290 (2015).

4. Mears, C., Wentz, F. J., Thorne, P. & Bernie, D. Assessing uncertainty in estimates of atmospheric temperature changes from MSU and AMSU using a Monte-Carlo technique. *J. Geophys. Res.* **116**, DOI:10.1029/2010JD014954 (2011).

5. Zou, C.-Z., Gao, M. & Goldberg, M. D. Error structure and atmospheric temperature trends in observations from the Microwave Sounding Unit. *J. Clim.* **22**, 1661–1681 (2009).

6. Zou, C.-Z. & Wang, W. Inter-satellite calibration of AMSU-A observations for weather and climate applications *J. Geophys. Res.* **116**, DOI:10.1029/2011JD016205 (2011).

7. Christy, J. R., Norris, W. B., Spencer, R. W. & Hnilo, J. J. Tropospheric temperature change since 1979 from tropical radiosonde and satellite measurements. *J. Geophys. Res.* **112**, D06102, DOI:10.1029/2005JD006881 (2007).

8. Karl, T. R., Hassol, S. J., Miller, C. D. & Murray, W. L. (eds). *Temperature trends in the lower atmosphere: Steps for understanding and reconciling differences. A Report by the U.S. Climate Change Science Program and the Subcommittee on Global Change Research.* National Oceanic and Atmospheric Administration, 164 pp. (2006).

9. Fu, Q., Johanson, C. M., Warren S. G. & Seidel, D. J. Contribution of stratospheric cooling to satellite-inferred tropospheric temperature trends. *Nature* **429**, 55–58 (2004).

10. Fu, Q. & Johanson, C. M. Stratospheric influences on MSU-derived tropospheric temperature trends: A direct error analysis. *J. Clim.* **17**, 4636–4640 (2004b).

11. Fu, Q. & Johanson, C. M. Satellite-derived vertical dependence of tropical tropospheric temperature trends. *Geophys. Res. Lett.* **32**, DOI:10.1029/2004GL02 2266 (2005).

12. Johanson, C. M. & Fu, Q. Robustness of tropospheric temperature trends from MSU Channels 2 and 4. *J. Clim.* **19**, 4234–4242 (2006).

13. Gillett, N. P., Santer, B. D. & Weaver, A. J. Quantifying the influence of stratospheric cooling on satellite-derived tropospheric temperature trends. *Nature*

**432**, DOI:10.1038/nature03209 (2004).

14. Kiehl, J. T., Caron J. & Hack, J. J. On using global climate model simulations to assess the accuracy of MSU retrieval methods for tropospheric warming trends. *J. Clim.* **18**, 2533–2539 (2005).

15. Santer, B. D. *et al.* Identifying human influences on atmospheric temperature. *Proc. Nat. Acad. Sci.* **110**, 26–33 (2013a).

16. Taylor, K. E., Stouffer, R. J. & Meehl, G. A. An overview of CMIP5 and the experiment design. *Bull. Amer. Meteor. Soc.* **93**, 485–498 (2012).

17. Eyring, V. *et al.* Long-term ozone changes ozone and associated climate impacts in CMIP5 simulations. *J. Geophys. Res.* **118**, 5029–5060 (2013).

18. Shindell, D. *et al.* Radiative forcing in the ACCMIP historical and future climate simulations. *Atmos. Chem. Phys.* **13**, 2939–2974 (2013).

19. Santer, B. D. *et al.* Comparing tropospheric warming in climate models and satellite data. *J. Clim.* **30**, 373–392 (2017).

20. U. S. Senate, 2015. Available online at: http://www.commerce.senate.gov/public/index.cfm/2015/12/data-or-dogma-promoting-open-inquiry-in-the-debate-over-the-magnitude-of-human-impact-on-earth-s-climate. "Data or Dogma? Promoting open inquiry in the debate over the magnitude of human impact on Earth's climate". Archived webcast of Hearing before the U. S. Senate Com-

mittee on Commerce, Science, and Transportation, Subcommittee on Space, Science, and Competitiveness, December 8, 2015 (Date of access: 28/03/2017).

**Supplementary Figure S1**: As for Fig. 1, but for maximally overlapping 18-year trends in near-global averages of corrected TMT. In ref. 19, the claim that "satellite data show no significant warming over the last 18 years"[20] was shown to be incorrect (at a stipulated significance level of 10% or better) in five out of six satellite TMT datasets. The trend significance assessment in ref. 19 considered satellite data ending in June 2016. An update of the analysis in ref. 19 (using TMT data through to December 2016 inclusive) reveals that in all six satellite datasets examined here, there is now significant global warming of the troposphere over the last 18 years (see Fig. S1C).

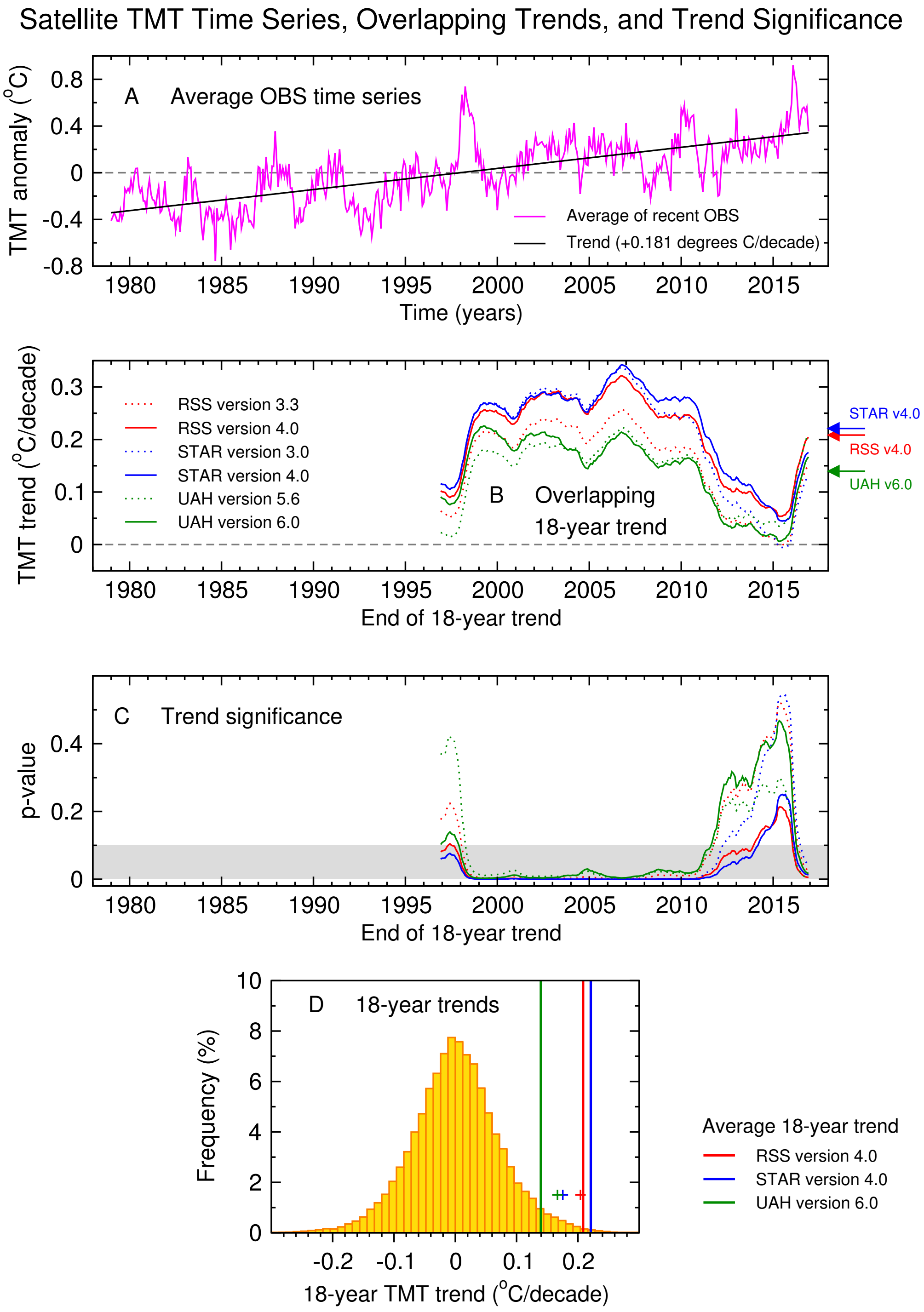


Supplementary Figure S1: Santer et al.

Supplementary Table 1: CMIP5 models used in this study.

| | **Model** | **Country** | **Modeling center** |
|---|---|---|---|
| 1 | ACCESS1.0 | Australia | Commonwealth Scientific and Industrial Research Organization and Bureau of Meteorology |
| 2 | ACCESS1.3 | Australia | Commonwealth Scientific and Industrial Research Organization and Bureau of Meteorology |
| 3 | BCC-CSM1.1 | China | Beijing Climate Center, China Meteorological Administration |
| 4 | BCC-CSM1.1(m) | China | Beijing Climate Center, China Meteorological Administration |
| 5 | CanESM2 | Canada | Canadian Centre for Climate Modelling and Analysis |
| 6 | CCSM4 | USA | National Center for Atmospheric Research |
| 7 | CESM1-BGC | USA | National Science Foundation, U.S. Dept. of Energy, National Center for Atmospheric Research |
| 8 | CESM1-CAM5 | USA | National Science Foundation, U.S. Dept. of Energy, National Center for Atmospheric Research |
| 9 | CMCC-CESM | Italy | Centro Euro-Mediterraneo per I Cambiamenti Climatici |
| 10 | CMCC-CM | Italy | Centro Euro-Mediterraneo per I Cambiamenti Climatici |
| 11 | CMCC-CMS | Italy | Centro Euro-Mediterraneo per I Cambiamenti Climatici |
| 12 | CSIRO-Mk3.6.0 | Australia | Commonwealth Scientific and Industrial Research Organization in collaboration with Queensland Climate Change Centre of Excellence |
| 13 | FGOALS-g2 | China | LASG, Institute of Atmospheric Physics, Chinese Academy of Sciences; and CESS, Tsinghua University |
| 14 | FIO-ESM | China | The First Institute of Oceanography, SOA |
| 15 | GFDL-CM3 | USA | NOAA Geophysical Fluid Dynamics Laboratory |
| 16 | GFDL-ESM2G | USA | NOAA Geophysical Fluid Dynamics Laboratory |

Supplementary Table 1: CMIP5 models used in this study (continued).

| | **Model** | **Country** | **Modeling center** |
|---|---|---|---|
| 17 | GFDL-ESM2M | USA | NOAA Geophysical Fluid Dynamics Laboratory |
| 18 | GISS-E2-H (p1) | USA | NASA Goddard Institute for Space Studies |
| 19 | GISS-E2-H (p2) | USA | NASA Goddard Institute for Space Studies |
| 20 | GISS-E2-H (p3) | USA | NASA Goddard Institute for Space Studies |
| 21 | GISS-E2-R (p1) | USA | NASA Goddard Institute for Space Studies |
| 22 | GISS-E2-R (p2) | USA | NASA Goddard Institute for Space Studies |
| 23 | HadGEM2-CC | UK | Met. Office Hadley Centre |
| 24 | HadGEM2-ES | UK | Met. Office Hadley Centre |
| 25 | INM-CM4 | Russia | Institute for Numerical Mathematics |
| 26 | IPSL-CM5A-LR | France | Institut Pierre-Simon Laplace |
| 27 | IPSL-CM5A-MR | France | Institut Pierre-Simon Laplace |
| 28 | IPSL-CM5B-LR | France | Institut Pierre-Simon Laplace |
| 29 | MIROC5 | Japan | Atmosphere and Ocean Research Institute (the University of Tokyo), National Institute for Environmental Studies, and Japan Agency for Marine-Earth Science and Technology |
| 30 | MIROC-ESM-CHEM | Japan | As for MIROC5 |
| 31 | MIROC-ESM | Japan | As for MIROC5 |
| 32 | MPI-ESM-LR | Germany | Max Planck Institute for Meteorology |

Supplementary Table 1: CMIP5 models used in this study (continued).

| | **Model** | **Country** | **Modeling center** |
|---|---|---|---|
| 33 | MPI-ESM-MR | Germany | Max Planck Institute for Meteorology |
| 34 | MRI-CGCM3 | Japan | Meteorological Research Institute |
| 35 | NorESM1-M | Norway | Norwegian Climate Centre |
| 36 | NorESM1-ME | Norway | Norwegian Climate Centre |

Supplementary Table 2: Start dates, end dates, and lengths ($N_m$, in months) of the 36 CMIP5 pre-industrial control runs used in this study. EM is the "ensemble member" identifier.*

| | Model | EM | Start | End | $N_m$ |
|---|---|---|---|---|---|
| 1 | ACCESS1.0 | r1i1p1 | 300-01 | 799-12 | 6000 |
| 2 | ACCESS1.3 | r1i1p1 | 250-01 | 749-12 | 6000 |
| 3 | BCC-CSM1.1 | r1i1p1 | 1-01 | 500-12 | 6000 |
| 4 | BCC-CSM1.1(m) | r1i1p1 | 1-01 | 400-12 | 4800 |
| 5 | CanESM2 | r1i1p1 | 2015-01 | 3010-12 | 11952 |
| 6 | CCSM4 | r1i1p1 | 800-01 | 1300-12 | 6012 |
| 7 | CESM-BGC | r1i1p1 | 101-01 | 600-12 | 6000 |
| 8 | CESM-CAM5 | r1i1p1 | 1-01 | 319-12 | 3828 |
| 9 | CMCC-CESM | r1i1p1 | 4324-01 | 4600-12 | 3324 |
| 10 | CMCC-CM | r1i1p1 | 1550-01 | 1879-12 | 3960 |
| 11 | CMCC-CMS | r1i1p1 | 3684-01 | 4183-12 | 6000 |
| 12 | CSIRO-Mk3.6.0 | r1i1p1 | 1651-01 | 2150-12 | 6000 |
| 13 | FGOALS-g2 | r1i1p1 | 201-01 | 900-12 | 8400 |
| 14 | FIO-ESM | r1i1p1 | 401-01 | 1200-12 | 9600 |
| 15 | GFDL-CM3 | r1i1p1 | 1-01 | 500-12 | 6000 |
| 16 | GFDL-ESM2G | r1i1p1 | 1-01 | 500-12 | 6000 |
| 17 | GFDL-ESM2M | r1i1p1 | 1-01 | 500-12 | 6000 |
| 18 | GISS-E2-H (p1) | r1i1p1 | 2410-01 | 2949-12 | 6480 |
| 19 | GISS-E2-H (p2) | r1i1p2 | 2490-01 | 3020-12 | 6372 |
| 20 | GISS-E2-H (p3) | r1i1p3 | 2490-01 | 3020-12 | 6372 |
| 21 | GISS-E2-R (p1) | r1i1p1 | 3981-01 | 4530-12 | 6600 |
| 22 | GISS-E2-R (p2) | r1i1p2 | 3590-01 | 4120-12 | 6372 |
| 23 | HadGEM2-CC | r1i1p1 | 1859-12 | 2099-12 | 2881 |
| 24 | HadGEM2-ES | r1i1p1 | 1859-12 | 2435-11 | 6912 |
| 25 | INM-CM4 | r1i1p1 | 1850-01 | 2349-12 | 6000 |
| 26 | IPSL-CM5A-LR | r1i1p1 | 1800-01 | 2799-12 | 12000 |

Supplementary Table 2 (continued): Information on the 36 CMIP5 pre-industrial control runs used in this study.

| Model | | EM | Start | End | $N_m$ |
|---|---|---|---|---|---|
| 27 | IPSL-CM5A-MR[§] | r1i1p1 | 1800-01 | 2068-12 | 3228 |
| 28 | IPSL-CM5B-LR | r1i1p1 | 1830-01 | 2129-12 | 3600 |
| 29 | MIROC5 | r1i1p1 | 2000-01 | 2669-12 | 8040 |
| 30 | MIROC-ESM-CHEM | r1i1p1 | 1846-01 | 2100-12 | 3060 |
| 31 | MIROC-ESM | r1i1p1 | 1800-01 | 2330-12 | 6372 |
| 32 | MPI-ESM-LR | r1i1p1 | 1850-01 | 2849-12 | 12000 |
| 33 | MPI-ESM-MR | r1i1p1 | 1850-01 | 2849-12 | 12000 |
| 34 | MRI-CGCM3 | r1i1p1 | 1851-01 | 2350-12 | 6000 |
| 35 | NorESM1-M | r1i1p1 | 700-01 | 1200-12 | 6012 |
| 36 | NorESM1-ME | r1i1p1 | 901-01 | 1152-12 | 3024 |

[*] See http://cmip-pcmdi.llnl.gov/cmip5/documents.html for further details.

[§] The `IPSL-CM5A-MR` control run has a large discontinuity in year 2069. We therefore truncated the `IPSL-CM5A-MR` control run after December 2068.
